# Variation in prognosis and treatment outcome in juvenile myoclonic epilepsy: a Biology of Juvenile Myoclonic Epilepsy Consortium proposal for a practical definition and stratified medicine classifications

**DOI:** 10.1093/braincomms/fcad182

**Published:** 2023-06-09

**Authors:** Guido Rubboli, Christoph P Beier, Kaja K Selmer, Marte Syvertsen, Amy Shakeshaft, Amber Collingwood, Anna Hall, Danielle M Andrade, Choong Yi Fong, Joanna Gesche, David A Greenberg, Khalid Hamandi, Kheng Seang Lim, Ching Ching Ng, Alessandro Orsini, BIOJUME Consortium, Lisa Strug, Naim Panjwani, Fan Lin, Danielle Andrade, Jana Zarubova, Zuzana Šobíšková, Cechovaz, Pracoviste, Michaela Kajsova, Guido Rubboli, Rikke S Møller, Elena Gardella, Christoph P Beier, Joanna Gesche, Maria Miranda, Inga Talvik, Pasquale Striano, Alessandro Orsini, Choong Yi Fong, Ching Ching Ng, Kheng Seang Lim, Kaja K Selmer, Marte Syvertsen, Pronab Bala, Amy Kitching, Kate Irwin, Lorna Walding, Lynsey Adams, Uma Jegathasan, Rachel Swingler, Rachel Wane, Julia Aram, Nikil Sudarsan, Dee Mullan, Rebecca Ramsay, Vivien Richmond, Mark Sargent, Paul Frattaroli, Matthew Taylor, Marie Home, Sal Uka, Susan Kilroy, Tonicha Nortcliffe, Halima Salim, Kelly Holroyd, Khalid Hamandi, Alison McQueen, Dympna Mcaleer, Dina Jayachandran, Dawn Egginton, Bridget MacDonald, Michael Chang, David Deekollu, Alok Gaurav, Caroline Hamilton, Jaya Natarajan Inyan Takon, Janet Cotta, Nick Moran, Jeremy Bland, Rosemary Belderbos, Heather Collier, Joanne Henry, Matthew Milner, Sam White, Michalis Koutroumanidis, William Stern, Mark P Richardson, Jennifer Quirk, Javier Peña Ceballos, Anastasia, Papathanasiou, Ioannis Stavropoulos, Dora Lozsadi, Andrew Swain, Charlotte Quamina, Jennifer Crooks, Tahir Majeed, Sonia Raj, Shakeelah Patel, Michael Young, Melissa Maguire, Munni Ray, Caroline Peacey, Linetty Makawa, Asyah Chhibda, Eve Sacre, Shanaz Begum, Martin O’Malley, Lap Yeung, Claire Holliday, Louise Woodhead, Karen Rhodes, Rhys Thomas, Shan Ellawela, Joanne Glenton, Verity Calder, John Davis, Paul McAlinden, Sarah Francis, Lisa Robson, Karen Lanyon, Graham Mackay, Elma Stephen, Coleen Thow, Margaret Connon, Martin Kirkpatrick, Susan MacFarlane, Anne Macleod, Debbie Rice, Siva Kumar, Carolyn Campbell, Vicky Collins, William Whitehouse, Christina Giavasi, Boyanka Petrova, Thomas Brown, Catie Picton, Michael O’Donoghue, Charlotte West, Helen Navarra, Seán J Slaght, Catherine Edwards, Andrew Gribbin, Liz Nelson, Stephen Warriner, Heather Angus-Leppan, Loveth Ehiorobo, Bintou Camara, Tinashe Samakomva, Rajiv Mohanraj, Vicky Parker, Rajesh Pandey, Lisa Charles, Catherine Cotter, Archana Desurkar, Alison Hyde, Rachel Harrison, Markus Reuber, Rosie Clegg, Jo Sidebottom, Mayeth Recto, Patrick Easton, Charlotte Waite, Alice Howell, Jacqueline Smith, Rosie Clegg, Shyam Mariguddi, Zena Haslam, Elizabeth Galizia, Hannah Cock, Mark Mencias, Samantha Truscott, Deirdre Daly, Hilda Mhandu, Nooria Said, Mark Rees, Seo-Kyung Chung, Owen Pickrell, Beata Fonferko-Shadrach, Mark Baker, Amy Whiting, Louise Swain, Kirsty O’Brien, Fraser Scott, Naveed Ghaus, Gail Castle, Jacqui Bartholomew, Ann Needle, Julie Ball, Andrea Clough, Shashikiran Sastry, Charlotte Busby Amit Agrawal, Debbie Dickerson, Almu Duran, Muhammad Khan, Laura Thrasyvoulou, Eve Irvine, Sarah Tittensor, Jacqueline Daglish, Sumant Kumar, Claire Backhouse, Claire Mewies, Julia Aram, Nikil Sudarsan, Dee Mullan, Rebecca Ramsay, Vivien Richmond, Denise Skinner, Mark Sargent, Rahul Bharat, Sarah-Jane Sharman, Arun Saraswatula, Helen Cockerill, David A Greenberg, Pasquale Striano, Rhys H Thomas, Jana Zarubova, Mark P Richardson, Lisa J Strug, Deb K Pal

**Affiliations:** Danish Epilepsy Centre, Filadelfia, Dianalund 4293, Denmark; Institute of Clinical Medicine, University of Copenhagen, Copenhagen 2200, Denmark; Department of Neurology, Odense University Hospital, Odense 5000, Denmark; Department of Research and Innovation, Division of Clinical Neuroscience, Oslo University Hospital, Oslo 0372, Norway; National Centre for Epilepsy, Oslo University Hospital, Oslo 1337, Norway; Department of Neurology, Drammen Hospital, Vestre Viken Health Trust, Oslo 3004, Norway; Department of Basic and Clinical Neurosciences, Institute of Psychiatry, Psychology and Neuroscience, King’s College London, London SE5 8AF, UK; MRC Centre for Neurodevelopmental Disorders, King’s College London, London SW1H 9NA, UK; Department of Basic and Clinical Neurosciences, Institute of Psychiatry, Psychology and Neuroscience, King’s College London, London SE5 8AF, UK; Department of Basic and Clinical Neurosciences, Institute of Psychiatry, Psychology and Neuroscience, King’s College London, London SE5 8AF, UK; Adult Epilepsy Genetics Program, Krembil Research Institute, University of Toronto, Toronto M5T 0S8, Canada; Division of Paediatric Neurology, Department of Pediatrics, Faculty of Medicine, University of Malaya, Kuala Lumpur 50603, Malaysia; Department of Neurology, Odense University Hospital, Odense 5000, Denmark; Abigail Wexner Research Institute, Nationwide Children’s Hospital, Columbus 43215, USA; Department of Neurology, Cardiff & Vale University Health Board, Cardiff CF14 4XW, UK; Division of Neurology, Department of Medicine, Faculty of Medicine, University of Malaya, Kuala Lumpur 50603, Malaysia; Institute of Biological Sciences, Faculty of Science, University of Malaya, Kuala Lumpur 50603, Malaysia; Department of Clinical and Experimental Medicine, Pisa University Hospital, Pisa 56126, Italy; Pediatric Neurology and Muscular Disease Unit, IRCCS Istituto ‘G. Gaslini’, Genova 16147, Italy; Department of Neurosciences, Rehabilitation, Ophthalmology, Genetics, Maternal and Child Health, University of Genova, Genova 16132, Italy; Newcastle upon Tyne NHS Foundation Trust, Newcastle upon Tyne NE7 7DN, UK; Translational and Clinical Research Institute, Faculty of Medical Sciences, Newcastle University, Newcastle upon Tyne NE1 7RU, UK; Department of Neurology, Second Faculty of Medicine, Charles University, Prague 150 06, Czech Republic; Motol University Hospital, Prague 150 06, Czech Republic; Department of Basic and Clinical Neurosciences, Institute of Psychiatry, Psychology and Neuroscience, King’s College London, London SE5 8AF, UK; MRC Centre for Neurodevelopmental Disorders, King’s College London, London SW1H 9NA, UK; School of Neuroscience, Institute of Psychiatry, Psychology and Neuroscience, King’s College, London SE5 8AF, UK; Program in Genetics and Genome Biology, The Hospital for Sick Children, Toronto M5G 1X8, Canada; Departments of Statistical Sciences and Computer Science and Division of Biostatistics, The University of Toronto, Toronto M5G 1Z5, Canada; Department of Basic and Clinical Neurosciences, Institute of Psychiatry, Psychology and Neuroscience, King’s College London, London SE5 8AF, UK; MRC Centre for Neurodevelopmental Disorders, King’s College London, London SW1H 9NA, UK; School of Neuroscience, Institute of Psychiatry, Psychology and Neuroscience, King’s College, London SE5 8AF, UK

**Keywords:** prognosis, classification, definition, juvenile myoclonic epilepsy, drug resistance

## Abstract

Reliable definitions, classifications and prognostic models are the cornerstones of stratified medicine, but none of the current classifications systems in epilepsy address prognostic or outcome issues. Although heterogeneity is widely acknowledged within epilepsy syndromes, the significance of variation in electroclinical features, comorbidities and treatment response, as they relate to diagnostic and prognostic purposes, has not been explored. In this paper, we aim to provide an evidence-based definition of juvenile myoclonic epilepsy showing that with a predefined and limited set of mandatory features, variation in juvenile myoclonic epilepsy phenotype can be exploited for prognostic purposes. Our study is based on clinical data collected by the Biology of Juvenile Myoclonic Epilepsy Consortium augmented by literature data. We review prognosis research on mortality and seizure remission, predictors of antiseizure medication resistance and selected adverse drug events to valproate, levetiracetam and lamotrigine. Based on our analysis, a simplified set of diagnostic criteria for juvenile myoclonic epilepsy includes the following: (i) myoclonic jerks as mandatory seizure type; (ii) a circadian timing for myoclonia not mandatory for the diagnosis of juvenile myoclonic epilepsy; (iii) age of onset ranging from 6 to 40 years; (iv) generalized EEG abnormalities; and (v) intelligence conforming to population distribution. We find sufficient evidence to propose a predictive model of antiseizure medication resistance that emphasises (i) absence seizures as the strongest stratifying factor with regard to antiseizure medication resistance or seizure freedom for both sexes and (ii) sex as a major stratifying factor, revealing elevated odds of antiseizure medication resistance that correlates to self-report of catamenial and stress-related factors including sleep deprivation. In women, there are reduced odds of antiseizure medication resistance associated with EEG-measured or self-reported photosensitivity. In conclusion, by applying a simplified set of criteria to define phenotypic variations of juvenile myoclonic epilepsy, our paper proposes an evidence-based definition and prognostic stratification of juvenile myoclonic epilepsy. Further studies in existing data sets of individual patient data would be helpful to replicate our findings, and prospective studies in inception cohorts will contribute to validate them in real-world practice for juvenile myoclonic epilepsy management.

## Teaser

Rubboli *et al*. provide evidence-based diagnostic criteria for juvenile myoclonic epilepsy based on clinical data from the BIOJUME Consortium and literature review. They propose a predictive model showing the prognostic value of absence seizures and female sex in predicting drug resistance and photosensitivity in females in reducing the odds of drug refractoriness.

## Graphical Abstract

**Graphical Abstract:**
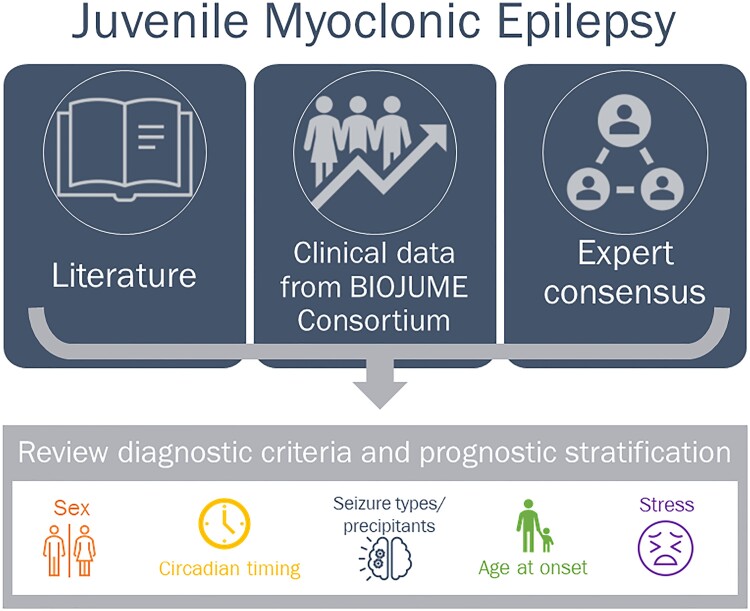

## Introduction

**Figure 1:**
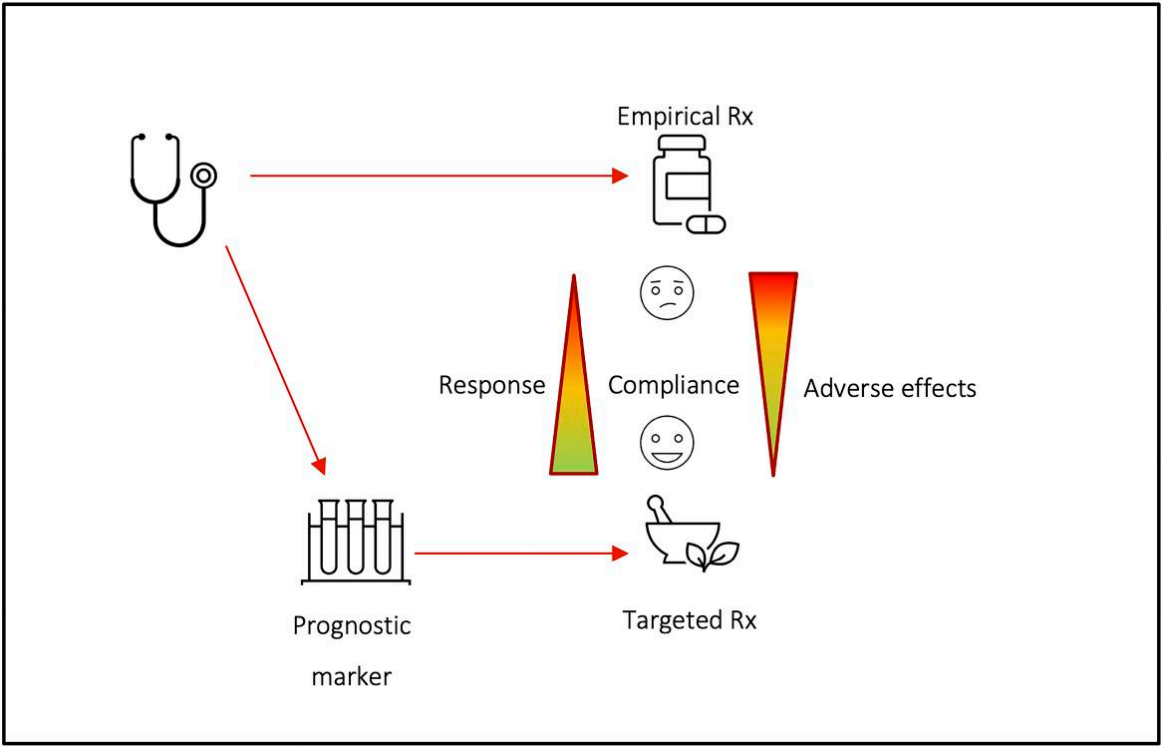
**Identification of clinical biomarkers by stratified medicine can pave the way to targeted treatments.** Stratified medicine adds a step to empirical medicine to associate a patient with a specific therapy that is more likely to be effective and/or safe. Central to the process is the identification of clinical biomarkers that differentiate subgroups of patients with differential treatment response, e.g. response or adverse effects. Based on Trusheim *et al*.^1^

Few diseases in medicine benefit from a prognostic model, and the epilepsies are no exception. Prognostic models are vital for the mission of stratified medicine, which can be summarized as identifying individuals with a condition who may profit from, or be harmed by, specific interventions.^1^ Accurate stratification is efficient at an individual and at a group level, permitting both personalization of treatment and smarter clinical trials design. Large-scale primary data sets and secondary meta-analyses of clinical trials and cohort studies offer the possibility of generating such models, testable for their utility in clinical practice (Figure 1).

Juvenile myoclonic epilepsy (JME), a common and archetypal idiopathic generalized epilepsy (IGE) syndrome, has proven challenging to define, thus rendering prognostic and outcome measures also difficult to delineate. None of the current epilepsy classification systems is focused on prognosis or outcome. A commonly used coding system like Systematized Nomenclature of Medicine–Clinical Terms (SNOMED-CT)^2^ denotes the syndrome of JME by the single code 62040001. In International Classification of Diseases 10th Revision (ICD-10),^3^ the JME code G40.B09 is subdivided into intractable versus not intractable (not defined) and then further subdivided by occurrence of status epilepticus (which is so rare as to be atypical in JME). Confusingly, the outcomes (intractability; status epilepticus) are comingled with the disease definitions. This system of coding is clearly inadequate and highlights the need for specificity of subclassification by epilepsy syndromes that goes beyond nosological purposes but that bears also diagnostic and prognostic significance. In 2011, an expert meeting^4^ nicknamed after its venue in Avignon failed to reach a consensus on the diagnosis and management of JME. Instead two separate classifications were proposed with limited clinical implications and without outlining possible prognostic indicators.^4^ That paper clearly highlights the variation in how the term JME was used globally, such as whether an abnormal MRI was acceptable and whether generalized abnormalities on the EEG were obligatory. In 2022, the International League Against Epilepsy (ILAE) Task Force on Nosology and Definitions released a position statement on definition of the IGE syndromes, including JME.^5^ The criteria for the definition of each syndrome relied on literature review until 2019, the most recent edition of the book ‘Epileptic Syndromes of Infancy, Childhood and Adolescence’,^6^ criteria listed in the ILAE website www.EpilepsyDiagnosis.org,^7^ and expert opinion from Task Force members. The consensus on the definition of each syndrome was achieved by a modified Delphi process.^8^ For JME, mandatory diagnostic criteria were myoclonic seizures and 3- to 5.5-Hz generalized spike wave (GSW) or generalized polyspike wave on EEG (even as retrieved historical data). As possible prognostic markers, the paper recognizes that a number of individuals and seizure-specific factors are associated with a tendency towards drug resistance, including the presence of absence seizures, psychiatric comorbidities, a prior history of childhood absence epilepsy (CAE), praxis-induced seizures and younger age at epilepsy onset.^5^

Progress on prognosis has been made through subclassification of JME.^9,10^ Although heterogeneity is widely recognized within epilepsy syndromes, confusion remains over the significance of variation in electroclinical features, comorbidities and treatment response.^11,12^

Here, we aim to show that with a predefined and limited set of mandatory features, variation in JME phenotype can be exploited for prognostic purposes. Our study was based on real clinical data collected by the Biology of Juvenile Myoclonic Epilepsy (BIOJUME) Consortium in concert with data from the published literature. The BIOJUME Consortium is a clinical genetic research project that includes the world’s largest cohort of subjects with JME, encompassing >900 cases.^13,14^ This cohort provides the possibility of gaining insights from an unprecedented large collection of clinical, EEG, behavioural and treatment data. For our analysis, we selected potential predictor variables that likely underlie clinical heterogeneity, and we focus specifically on easily measured stratifying variables associated with clinically relevant influence on prognosis, treatment outcome or adverse drug effects.

## Materials and methods

We approached this task by separately evaluating diagnostic criteria, prognosis, predictors of drug resistance and adverse events.

### Diagnostic criteria for definition

We took the diagnostic criteria of the Avignon meeting as our starting point: these included myoclonic jerks and their timing, age of seizure onset and intelligence.^4^ We reviewed relevant literature using search terms (‘juvenile myoclonic epilepsy’ OR ‘Janz syndrome’ OR ‘idiopathic generalized epilepsy’ OR ‘genetic generalized epilepsy’) AND (‘late onset’). For intelligence, we performed a systematic search in the PubMed database (search string: ((epilepsy, juvenile myoclonic[MeSH Terms]) OR (juvenile myoclonic epilepsy[Title/Abstract])) AND ((intell*[Title/Abstract]) OR (neuropsych*[Title/Abstract]))). We also analysed primary data in the BIOJUME data set concerning morning predominance of myoclonic seizures. We questioned accepted generalizations eventually leading to restrictive thinking about JME diagnosis. In the field of logic, a ‘fallacy of defective induction’ is a conclusion based on weak (biased or insufficient) premises; e.g. all swans are white, *ergo* black swans cannot be swans. We also tested some of the prevailing assumptions about phenotype in JME by examining ‘counterfactual’ arguments. A counterfactual argument asks what would be the logical conclusion if the opposite was true; e.g. if black swans are not swans, what are they?

The BIOJUME Consortium recruited >900 participants from 58 sites across nine countries.^14^ Briefly, BIOJUME eligibility criteria were based on Avignon Class II (Table 1), and all phenotypes were reviewed by a panel of seven expert clinicians (C.P.B., K.H., D.K.P., M.R., G.R., M.S., and RHT). To explore the importance of a circadian entrainment of seizures, we compared participants with predominant morning seizures with those who reported no morning predominance for distribution of sex, age of onset, absence seizure history, history of triggered seizures, antiseizure medication (ASM) resistance, generalized and polyspike wave EEG features, photoparoxysmal response (PPR) and impulsivity score [measured with Barratt Impulsiveness Scale (BIS), versions BIS-Brief and BIS-11].^13^ We then generated a multivariable model of morning predominance incorporating sex and significant variables from univariate comparison, calculating odds ratios with 95% confidence intervals and exact *P*-values. Five authors (G.R., C.P.B., K.K.S., M.R.S., and D.K.P.) then summarized the overall evidence on diagnostic criteria and presented conclusions to the wider authorship.

**Table 1:** Avignon and ILAE draft criteria versus proposed BIOJUME criteria

	Avignon Class I	Avignon Class II	ILAE Task Force	BIOJUME
**Mandatory criteria**	Myoclonic jerks without loss of consciousness	Myoclonic jerks without loss of consciousness	Myoclonic seizures	Myoclonic jerks
	Myoclonic jerks exclusively on or after awakening	Myoclonic jerks predominantly on or after awakening		Myoclonic jerks timing **variable**
	Generalized EEG abnormalities	Generalized EEG abnormalities	Generalized EEG abnormalities	Generalized EEG abnormalities
	Age of onset 10–25 years	Age of onset 6–25 years	Age of onset 8–40 years	Age of onset ≥6 years **increases probability** of JME
	Normal intelligence	No mental retardation or deterioration	Exclude moderate to profound ID	Intelligence **conforms to population distribution**

Bold characters highlight distinct features of the BIOJUME diagnostic criteria as compared with previous diagnostic proposals.

### Prognosis and predictors of ASM resistance

For prognosis, we evaluated predictors of the specific outcomes of mortality and seizure remission; where JME-specific information was lacking, we imputed findings from the IGEs or epilepsy in general. We reviewed relevant literature, including a recent comprehensive publication of the BIOJUME Consortium^14^ and a meta-analysis,^15^ regarding potential predictors of ASM resistance: sex, age of onset, absence seizures, morning predominance of myoclonic seizures, stress-related seizures, PPR and EEG features. We used combinations of MeSH search terms in the PubMed database, unrestricted by year of publication or language: (‘juvenile myoclonic epilepsy’ OR ‘Janz syndrome’) AND humans AND (prognos* OR predict* OR biomarker OR outcome OR mortality OR epidemiology OR remission) AND ((antiseizure OR antiepileptic) AND (drug OR medication)) AND (*resistan* OR refractory OR intractable); sudden unexpected death in epilepsy (SUDEP): (‘generalized epilepsy’) AND (SUDEP) NOT (‘baboon’ OR ‘mouse’). Abstracts were then screened for relevance.

### Predictors of adverse drug events

We evaluated predictors of selected serious adverse drug events associated with the three most commonly prescribed ASMs in JME: valproate, weight gain; lamotrigine, rash; and levetiracetam, psychiatric adverse events. Because these drugs are not limited in their use to epilepsy, we did not specify a disease. We first searched PubMed using MeSH search terms: (levetiracetam [tiab] AND (adverse OR safety OR tolerability) [tiab] AND (psych* OR behav*)) OR f(valpro* [tiab]) AND (‘weight gain’ OR obesity OR overweight OR BMI OR metabolic) OR (lamotrigine AND (rash OR exanthema OR ‘Stevens-Johnson syndrome’ OR toxic epidermal necrolysis OR ‘drug rash with eosinophilia and systemic symptoms’)).

We then analysed data on impulsivity in JME for association with self-reported adverse psychiatric events to levetiracetam,^13^ calculating the predictive properties of a BIS-Brief cut-off score of ≥21. We also conducted a multiple logistic regression analysis in the same data set of self-reported valproate associated excessive weight gain with the following variables in the model: sex, absence seizure frequency, myoclonic seizure frequency, morning predominant seizures, BIS-Brief score and log of body mass index (BMI).

### Statistical analysis

Statistical analysis was carried out on SPSS statistics (version 25). Data on statistical analysis reported in Tables 3 and 4 are reproduced from Shakeshaft *et al*.^13^

## Results

### Diagnostic criteria for definition

#### Myoclonic jerks

In Dieter Janz’s description of JME in 1957,^16^ the defining seizure type was myoclonic seizures, which he called ‘impulsive petit mal’, referring to the sudden, impulse-like movement, usually proximally in the upper extremities. The two papers introducing JME to the English-speaking world also included it as part of the definition in 1984 and 1989 as did the 1989 ILAE Proposal.^16-18^ However, at the 2011 Avignon meeting, two of 14 experts stated that they did not consider myoclonic jerks mandatory for the diagnosis of JME^4^; the report did not elaborate further.

We could find no evidence in the literature to justify a change from the first definitions specifying myoclonic seizures as a critical feature for diagnosis. We speculate that concerns about misclassification might have been an issue at the Avignon meeting: patients who have poor recall of subtle jerks or who have been treated after a first generalized tonic–clonic seizure (GTCS) may be diagnosed as epilepsy with generalized tonic–clonic seizures alone (EGTCSA). Misclassification might occur also in patients in whom absence seizures predate myoclonus; therefore, in teenaged years, myoclonus is not the main seizure type, or in patients in whom myoclonus responds well to ASMs, and so there is a time where only occasional convulsive seizures, but no myoclonus, are reported. These concerns are not a reason to exclude myoclonic jerks from the definition of JME. Here, we align with the ILAE position statement^5^ that a history of myoclonic seizures is in fact mandatory for the diagnosis of JME.

#### Age of seizure onset

One major operational challenge remains the definition of the ‘age of onset’. It could be defined as the time of diagnosis, which is usually after the first GTCS. Given that myoclonic seizures precede GTCS by an average of 1–3.3 years,^13,19^ the age at first motor seizure may be more appropriate^20,21^ but then does not account for the subgroup of patients with CAE later developing JME.^22^ Sometimes the ‘age of onset’ of epilepsy might be difficult to determine in older patients with a single GTCS who do not recall myoclonus in adolescence.^23^ On the assumption that JME is a neurodevelopmental disorder, an upper maximal age of onset appears plausible.^24^ Conversely, as a polygenic disease^25^ with variable penetrance and a high incidence of generalized EEG changes amongst asymptomatic relatives,^26^ one may assume a variability in both genetic liability and environmental (seizure-provoking) factors allowing for the possibility of late (>25 years) first-time seizures.

The Avignon meeting proposed an age of onset between 10 and 25 years as Class I criterion or between 6 and 25 years as Class II criterion for the diagnosis of JME.^4^ The ILAE position statement permits an onset between 8 and 40 years, with a warning that an age of onset before 8–9 years or between 25 and 40 years should prompt one to reconsider the diagnosis (Table 1).^5^ There is broad consensus in the literature about the typical age of first GTCS in JME, which lies between 12 and 18 years in ∼75% of patients.^13,19,27-29^ However, evidence for exclusionary upper and lower age limits is sparse, and many cohorts included patients with an age of myoclonus onset below 6 or above 25.^27-29^

The existence of late-onset IGE and IGE with phantom absences as distinct entities remains controversial.^21,23,30,31^ Suffice to say, late-onset JME is extremely rare. Reichsoellner *et al*.^31^ showed that 28/429 IGE patients (5.7%) had late seizure onset, including two with JME (0.5%). JME mimics, including progressive myoclonus epilepsies, in adults are usually accompanied by additional neurological symptoms like dementia or ataxia^12,32^ (Supplementary Table 1), and true misdiagnosis is uncommon. The same applies to patients with debut before the age of 6. Although there is a broad range of genetic conditions associated with myoclonic epilepsy in children <6 years, progression of neurological/cognitive deficits and seizure severity^33^ usually exclude the diagnosis of JME. In the BIOJUME data set, the lower age of onset was constrained at 6 years, but the phenotyping committee diagnosed JME in participants with age of onset 6–37 years in females and 9–40 years in males.^13^

#### Intelligence

The observation of an impaired cognition and intelligence was not explicitly included in the early definitions, nor as a diagnostic criterion for JME in early papers or the ILAE definition of 1989.^17^ In the Avignon paper,^4^ intelligence was included as a criterion in both the proposed classes of diagnostic criteria (Table 1): in Class I, the criterion was ‘normal intelligence’ and in Class II ‘no mental retardation or deterioration’. Although not specifically discussed in the paper, the basis for proposing these criteria was that eight out of the 14 experts believed that ‘abnormal cognition’ was not allowed as part of the JME diagnosis. The recent ILAE position statement admits that ‘mild intellectual disability’ is observed in some patients; however, this clinical feature is considered an alert that should lead to consider alternative diagnoses.^5^

The question of whether intelligence needs to be normal in JME, and the scientific basis for having normal intelligence as an inclusion criterion, is not well addressed in the existing literature, and it is not further elaborated in the recent ILAE Position Paper.^5^ A review by Ratcliffe *et al.*^32^ addressing cognitive function in IGEs finds that the intelligence quotient (IQ) in JME is consistently reported to be within normal range and only slightly lower than IQ in healthy controls. None of the identified 124 papers in our literature search included patients below an IQ of 70. Thus, these results exemplify the inductive fallacy: namely, most people with JME have normal intelligence *ergo* normal intelligence defines JME. If we examine the counterfactual argument, then we have to ask first, whether not only those below the normal (95%) range of intelligence are excluded from the diagnosis but also those above the normal range; and second, if individuals with the typical features of JME in the context of intellectual disability do not have JME, then what do they have? This fallacy is likely to have been amplified by studies that focus on the initial diagnosis and concerns about missing progressive myoclonus epilepsies, without a commensurate attention on mature longitudinal studies. Intellectual disability, impaired neurocognitive functions, affected behavioural phenotypes and psychiatric symptoms are often reported and discussed as overlapping features; however, they might represent endophenotypes with shared aetiological factors. Several of the identified genetic susceptibility variants of JME are located in genes or regions inferring susceptibility also to other neurodevelopmental disorders (e.g. *GABRA1*, *EFHC1*, and *BRD2*),^34^ suggesting common pathological mechanisms. Recently, copy number variants contributing to both intellectual impairment and IGE have been identified (such as microdeletions at 15q13.3, 15q11.2, and 16p13.11),^35^ supporting the concept that it is possible to diagnose an IGE syndrome in patients with intellectual disability. In addition, recent studies applied advanced MRI technology to reveal subtle brain developmental abnormalities in JME, leading to pathophysiological hypotheses potentially explaining a range of clinical symptoms of JME, including impaired cognitive functions.^25,36^ As such, the view of JME as a disorder where inherent neurodevelopmental factors may also contribute to the phenotype and where JME is part of a much wider disease spectrum than the traditional inclusion criteria embrace may uncover key pathological mechanisms of JME. Such an expanded phenotypic view would certainly bring some challenges to both clinical and research classifications and perhaps prompt a subclassification of endophenotypes to enable clinical studies of well-defined and homogeneous entities. However, it may also enable the revelation of other hitherto unknown aspects of JME, which have remained concealed due to the limitations of the existing diagnostic criteria.

#### Morning predominance of myoclonic seizures

Diurnal variability in cortical excitability in individuals with IGE, with cyclic increases in the morning, has been elegantly demonstrated using transcranial magnetic stimulation–electroencephalography (TMS-EEG) methods.^37,38^ The broader amplitude of the paroxysms in the frontal regions in JME^39^ suggests a role of the frontal cortex in leading generalized discharges.^40^ Interestingly, some thalamic nuclei known to modulate functional states such as sleep and wakefulness (e.g. intralaminar nuclei and the reticular nucleus) are directly connected to the frontal cortex. Since the time of awakening is a critical condition for the activation of the epileptic discharges, the evidence that these nuclei play a role in spike-wave generation in experimental models of generalized epilepsies^41^ may suggest their involvement also in provoking myoclonus in JME. In addition, animal studies suggest that feedback loops of *Clock* proteins, amongst several other factors, influence this circadian variability as, in their absence, fluctuations in cortical excitability are diminished.^42^ In the other direction, epilepsy (and many other neuropsychiatric disorders), stress, inflammation, xenobiotics and genetic/epigenetic factors may alter the temporal expression and amplitude of *Clock* genes, leading to disrupted circadian rhythms in sleep and cortisol,^42^ possibly explaining the failure to exhibit morning peaks in cortical excitability in some patients with IGE.

The Avignon paper^4^ emphasizes the exclusive (narrow classification) or predominant (broad) timing of myoclonic seizures on awakening. It is difficult to find data on true circadian seizure distribution in JME because most studies since 1989 use this feature as an inclusion criterion, possibly leading to a circular reinforcement of the definition. This may be an example of the ‘inductive fallacy’, assuming that because the majority of individuals show awakening seizures, and ‘ignoring’ those who do not, that it is an invariant feature and therefore a mandatory diagnostic criterion. However, amongst the earliest descriptions of JME, Janz^20^ cites two studies in which an awakening predominance varied between 52% and 74%, and his own work acknowledges that such a pattern is not always seen. Subsequent studies report >80% awakening myoclonic seizures, and slightly less for tonic–clonic seizures, even when explicitly using ‘awakening’ seizures as an inclusion criterion (Table 1). In the recent ILAE position statement, myoclonic seizures are reported to occur ‘most commonly’ within the first hour after awakening.^5^ One study suggested that the lack of the awakening pattern was associated with praxis induction^43^; and it has also been suggested that awakening myoclonic and ‘grand-mal’ seizures may be differently inherited from those occurring at random times of day.^44^ When we look at the counterfactual argument, then clearly when myoclonic seizures are the predominant seizure type but not predominantly on awakening and other features (age of onset and EEG) are typical, there is no other diagnostic possibility than JME (Tables 2 and 3).

**Table 2:** Previous observations of awakening predominant seizures in JME

Authors	Inclusion criteria	Awakening MCJ	Awakening GTC
Simonsen 1975, cited in Janz^20^	Not stated	52%	NA
Van Heycop ten Ham 1982, cited in Janz^20^	Not stated	74%	NA
Janz^20^	Not stated	95%	NA
Clement *et al*. ^45^	Not stated	8/10 (80%)	6/9 (67%)
Panayiotopoulos^46^	‘Unequivocal clinical evidence of generalized seizures with myoclonic jerks **mainly on awakening**’	56/66 (88%)	11/16 (69%)
Murthy^47^	‘Clinical evidence of generalized seizures with myoclonic jerks mainly **on waking**’	112/120 (93.3%)	87/120 (78.4%)
Dhanuka^48^	‘Unequivocal clinical evidence of generalized seizures with myoclonic jerks **on awakening**’	24/24 (100%)	NA
Uchida^43^	All had unequivocal diagnosis of JME based on ‘electroclinical characteristics, including normal physical and neurological examinations, brain imaging and generalized 4- to 6-Hz spike or polyspike-wave complexes.’^7,42^	No Photic Induction 23/25 (92%) Photic induction 12/20 (60%)	

Bold characters highlight seizures occurrence in relation to the sleep-wake cycle.

MCJ, myoclonic jerks; GTC, generalized tonic–clinic seizures.

**Table 3:** Demographic and clinical differences in 588 JME patients with and without morning predominant myoclonic seizures in the BIOJUME data set

Feature comparison in morning entrainment patterns			
Variable	Morning predominant	Not morning predominant	*P*-value
Sex female	348/537 (64.8%)	74/126 (58.7%)	0.202
Age JME onset years (95% CI)	16.66 (15.99–17.86)	16.93 (16.33–17.07)	0.588
Absence seizures history	214/525 (40.8%)	65/124 (52.4%)	**0**.**018**
Triggered seizures	274/500 (54.8%)	69/118 (58.5%)	0.470
ASM resistance	119/371 (32.1%)	33/100 (33%)	0.861
EEG gen spike wave	254/537 (47.3%)	82/126 (65.1%)	**<0**.**001**
EEG polyspike wave	358/537 (66.7%)	72/126 (57.1%)	**0**.**044**
EEG photoparoxysmal response	165/450 (36.7%)	40/110 (36.3%)	0.953
BIS-Brief score (95% CI)	17.47 (17.02–17.92)	18.76 (17.77–19.75)	**0**.**012**

Logistic regression of morning predominant seizures			
Variable	Odds ratio (95% CI)	Z	*P*-value
Absence seizures	0.65 (0.43–0.99)	−1.99	**0**.**047**
EEG gen spike wave	0.42 (0.24–0.72)	−3.12	**0**.**002**
EEG polyspike wave	0.88 (0.51–1.51)	−0.48	0.634
Sex female	1.50 (0.97–2.31)	1.82	0.069
Age JME onset	1.04 (0.98–1.12)	1.33	0.184

Values in bold are statistically significant at the 5% level.

In the BIOJUME data set, 537/663 (81%) exhibited morning predominant myoclonic jerks. We investigated whether individuals with and without this feature differed significantly in demographic, clinical or prognostic features, i.e. whether awakening versus non-awakening predominant seizures represented a disease different to JME. We found no difference in sex or age distribution, but those lacking morning predominance were more likely to experience absence seizures; there was no difference in photosensitivity (Table 3), no association with triggers and importantly no difference in ASM resistance. In a multiple logistic regression model including sex, age of onset, absence seizures and EEG patterns, we confirmed that morning predominance is independently negatively associated with absence seizures [odds ratio (OR) 0.65; 95% confidence interval (CI) 0.43–0.99] and lack of GSW on EEG (Table 3). We found that individuals lacking morning predominance reported higher trait impulsivity, which may have been confounded by absence seizure frequency.^13^

The best evidence from these data is that absence seizures and EEG patterns are the most important influences on awakening patterns of seizures in JME. What we do not know is whether patients change in their circadian seizure patterns over time or whether a pattern of morning predominance is established from the onset—this would require a prospective longitudinal study.

##### Conclusion

We confirm myoclonic seizures as a mandatory diagnostic criterion for JME because of the lack of contrary evidence (Table 1). It is difficult to set age of onset criteria. However, definite cases with onset between the ages of 6 and 40 years have been agreed upon in our BIOJUME data set, thus slightly deviating from the ILAE position statement that admits a lower age limit not inferior to 8 years.^5^ We do not specify an intelligence range in our proposed definition, admitting an intelligence range conforming to population distribution. Future studies addressing the true IQ distribution of JME may reveal whether the degree of cognitive affection in JME harbours a prognostic value, also in terms of response to treatment, which should be included in a prognostic classification. Last, taking neurobiological, historical and empirical evidence together, we propose that morning predominance be considered a variable, not mandatory, feature in the definition of JME that is irrelevant to prognostic classification.

### Prognosis

#### Mortality

Premature mortality is recognized across epilepsy in general and varies by aetiology and between high- and low-income countries.^49^ A systematic review of 56 population and hospital-based epilepsy cohorts show that intractable epilepsy, symptomatic epilepsy, generalized seizures, brain tumours and ischaemic heart disease as causal factors and later studies accord with these findings.^50,51^ Comorbidity, especially neurological, male sex and race/ethnicity were associated with premature mortality amongst US Medicare patients^52^; demographic and clinical codes also accurately predicted risk of death in epilepsy patients from US insurance data.^53^ UK data confirm the importance of social deprivation as a risk factor not only for premature mortality in epilepsy but also for intellectual disability.^54^ The North American SUDEP Registry (NASR) suggested a risk higher than expected in patients with IGE (IGE accounted for one fourth of SUDEP cases in NASR), an association that awaits independent confirmation given the low number of events.^55^ Premature death or SUDEP seems to be rare in JME. Out of a series of 170 consecutive JME, only two patients with possible or probable SUDEP were reported, both cases suffering from severe mental disorders.^56^ Few other cases who died probably of SUDEP have been described, either in subjects not taking antiepileptic treatment or with well-controlled epilepsy.^57,58^

#### Seizure remission

IGEs in general have a better treatment outcome than symptomatic epilepsies,^15,59^ but the difference is not stark. The 1-year seizure freedom rate for people aged 9–33 years with IGE was 68.1%, compared with 62.5% for focal epilepsies,^60^ with some authors reporting much lower rates.^61^ It is often repeated that ‘80% of people with IGE respond to ASM’,^5^ but is that still the case when women of child-bearing potential are not to be prescribed valproate?^59^ There are very few studies either of people with untreated IGE or with a prospective design. Retrospective studies in limited patients series show that seizure remission is possible for many.^62,63^ Febrile seizures and epileptiform runs >3 s are suggested predictors of poorer long-term outcome in JME,^60,64^ whilst treatment with valproate and longer seizure freedom prior to ASM withdrawal are associated with higher chance of remission in treated cohorts.^65-67^ In the JME subclassification of Martinez-Juarez *et al*.,^9^ 58% of ‘classic JME’ (‘patients with adolescent onset of myoclonic, tonic–clonic and clonic–tonic–clonic seizures with or without rare-to-infrequent absences’) achieve seizure freedom on ASM therapy and 5% off medication, whilst in the group of CAE evolving to JME, only 7% were seizure free. Baykan *et al*.^57^ reported a benign course in about two-thirds of a cohort of 48 JME patients, with remission or marked alleviation of myoclonia for >8 years after patients achieved the age of ∼33 years, suggesting that in a significant proportion of patients, myoclonic seizures have a tendency towards remitting or becoming milder with age. Similar results were observed in a retrospective study on 61 JME patients who were followed for a mean duration of ∼29 years; 65% of patients had a 5-year remission with a mean age at the last seizure of 27.4 years.^68^ One-third of the seizure-free patients attempted ASM withdrawal and ∼50% of them relapsed. In contrast with these findings, another study^61^ reported a 2-year remission rate only in 22% of subjects in a cohort of 145 JME patients, with twice as high relapse rates in patients attempting drug withdrawal as compared with those who continued their ASM regimen. Finally, a recent study^69^ investigated in a JME cohort the 4-year remission for all seizure types starting within 2 years (early sustained remission) or after 2 years (delayed sustained remission) since the initiation of ASM intake. Four-year seizure remission was observed in 67.3% subjects, in line with previous studies.^57,68^ Early sustained remission was achieved by 59.2% patients. Spontaneous seizure relapse after 4-year remission occurred in 15.7% of patients with early sustained remission and in 35.5% of those with delayed sustained remission. Catamenial seizures and earlier age at epilepsy onset significantly predicted delayed sustained remission. These findings suggest that a positive response to the first ASM that leads to an early seizure remission can predict a favourable long-term seizure outcome.

### Predictors of drug response

#### ASM resistance

The literature search yielded 923 studies (search date 11 February 2022), but only two relevant recent papers had not been included in the 2019 meta-analysis of Stevelink *et al*.^15^

##### Age of onset

Data from the BIOJUME consortium show that myoclonus onset before the age of 12 years was associated with drug resistance only in females; the age stratification accords with other studies.^15^

##### Sex

**Figure 2:**
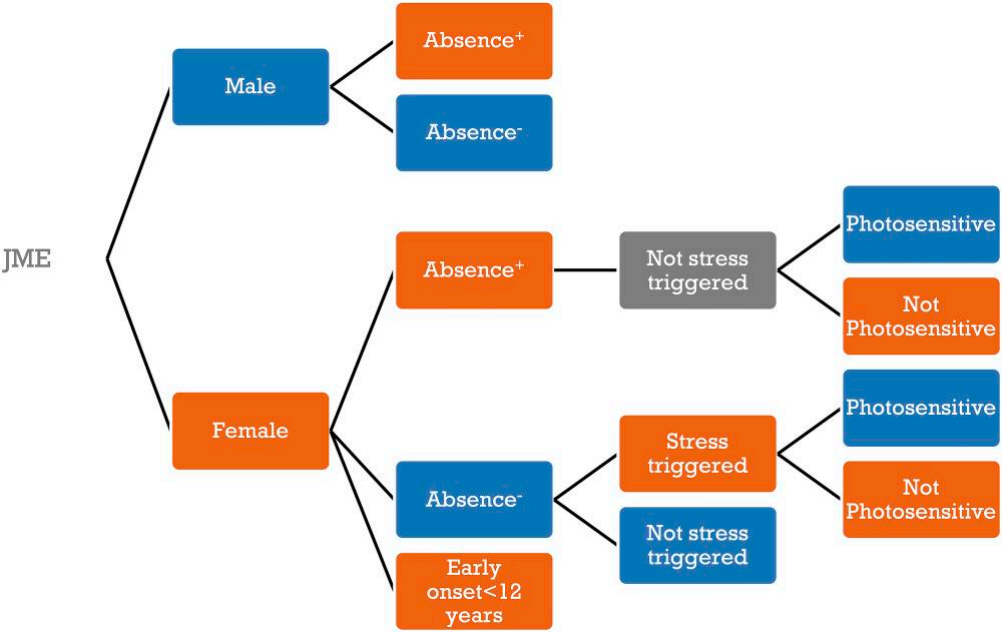
**Proposed predictive model of ASM resistance in JME based on Shakeshaft *et al*.** ^14^ At each stratum, blue denotes better prognosis, orange worse prognosis, and grey denotes neutral effect on outcome.

In the BIOJUME data set, there are significant treatment outcome differences between males and females with JME, necessitating sex stratification in management.^14^ Absence seizures strongly predict ASM resistance in both sexes, but, in females only, stress-precipitated seizures and catamenial seizures are associated with ASM resistance and PPR with seizure freedom. Females with both absence seizures and ‘stress-related’ precipitants (defined as physiological states that influence neurobiological stress circuits, including stress itself, sleep deprivation, menstrual cycle and concentration efforts, discussed in Shakeshaft *et al*.^14^) constitute the prognostic subgroup in JME with highest prevalence of ASM resistance (49%) compared with females with neither (15%); see also below. A proposed prognostic classification based on these findings is illustrated in Fig. 2.

Lack of sex stratification has perhaps contributed to inconsistent evidence for differential prognosis in females in previous studies.^70^ The relationship between sex and prognosis is nuanced however, suggesting a hierarchy of factors: stress precipitants only increase the odds of ASM resistance in females ‘without’ absence seizures, whilst photosensitivity decreases the odds of ASM resistance in females ‘regardless’ of absence seizures.^14^

##### Absence seizures

The strong effect of absence seizures on ASM resistance reported multiple times^71^ is strongly associated with trait impulsivity in JME.^13^ Absence seizures, visual sensitivity and stress-related precipitants in JME may not just be clinical features, but the instantiation of separate seizure susceptibility networks with their own distinct effect on seizures and behavioural outcomes,^72^ which may suggest completely new therapeutic approaches such as circuit-specific therapy.^73,74^

##### Triggered seizures

In the BIOJUME data set, around half of all individuals with JME report triggered seizures, with a slight female excess (59% versus 50%).^14^ Moreover, this subgroup reports a large proportion (median 70%) of their seizures to be triggered, with one in five estimating that ‘all’ their seizures are precipitated. In previous analysis, we saw that just five triggers accounted for >80% of the total: sleep disturbance, stress, alcohol, visual/lights stimuli and menstrual cycle, which are well-known in JME^75,76^ and other epilepsies.^77-79^ We found no association of any trigger with seizure control in males but a marked difference in trigger/seizure control associations, depending on whether females experience absence seizures.

##### Photoparoxysmal response

PPR recorded in the EEG lab can be seen in up to 90% of untreated subjects with JME according to the ILAE position statement.^5^ However, precise estimates can be marred by the fact that photosensitivity can fluctuate across days or weeks, can be lost with age, can be modified by treatment, and can depend on laboratory stimulation procedures.^80^ PPR and self-reported photosensitivity are also more common in females, consistent with previous studies showing a female excess of 1.5–2.^81-83^ BIOJUME data analysis shows a strong relationship between self-reported triggered seizures and PPR, with 71% of those with PPR reporting triggered seizures.^14^ More specifically, 63% of those who report light/visual patterns as a trigger also had PPR, and 24% of those with PPR reported light/visual patterns as a trigger.^14^ A lesser degree of association was observed between the presence of PPR and other precipitants: stress, sleep disturbance, praxis and concentration in females, and with alcohol in males.^14^ This finding suggests that failure to conduct a sex-stratified analysis may explain the lack of overall association in a recent meta-analysis that found a protective effect of PPR on seizure freedom in four out of five studies.^70^ One possibility is that the component of seizure susceptibility mediated via visual pathway hypersensitivity^76,84,85^ is effectively treated by current ASMs, a hypothesis we are unable to test in the BIOJUME data but that merits further investigation.

##### Psychiatric comorbidity

Coexistent psychiatric or personality disorders are associated with ASM resistance^10,15,27,57,86-88^ although in the absence of prospective studies, the direction of this association is uncertain.^89^

##### EEG biomarkers

Focal EEG features have been variably defined in the literature (e.g. lateralized GSW, asymmetric amplitude of GSW, focal spikes or focal slowing),^87^ but prognostic analyses have not included known predictors in multivariable analyses.^90,91^ Hence, although focal EEG features have been proposed as a predictor of ASM resistance and refuted by others,^60,92^ confounding by other variables is possible, and hence their utility is unproven. However, other EEG features^93^ such as generalized polyspike train during sleep have been validated in multivariable analyses and replicated in IGE cohorts, including JME.^93,94^ Differences in functional network topology computed from EEG may also provide biomarkers of drug resistance.^95^ However, the assessment of specific EEG features, or prolonged recording, is not a routine part of the neurophysiological practice and therefore may be impractical for a simple classification scheme.

#### ASM adverse events

Our literature search identified 470 articles related to levetiracetam (*n* = 20 relevant after reviewing abstracts), 522 for valproate (*n* = 19) and 381 for lamotrigine (*n* = 34). Adverse effects to the three most common ASMs used in IGE appear to correlate with efficacy: valproate, levetiracetam and lamotrigine in declining order.^59,96-98^ Since the probability of failure with the first ASM is so high,^59^ due to either lack of efficacy or intolerable adverse effects, there is an urgent need for predictive tools to avoid iatrogenic morbidity. Some adverse effects are shared across ASMs, for example somnolence and dizziness, whilst others are more specific such as paradoxical seizure exacerbation, or skin rash with lamotrigine; psychiatric disturbance with levetiracetam^99^; and weight gain or polycystic ovary syndrome with valproate.^100^

##### Levetiracetam

Attempts have been made to develop predictors of psychiatric adverse effects to levetiracetam.^13,101,102^ The most recent of these showed that people who had levetiracetam-induced psychosis had an increased polygenic risk score for schizophrenia than those who did not.^103^ HLA-A*1101 is enriched amongst Korean epilepsy patients with psychiatric adverse events to levetiracetam.^104^ Genetic variation in dopaminergic activity has also been suggested as an association.^102^

Trait impulsivity is independently associated with the risk of an adverse psychiatric event on levetiracetam, as shown by a score on the BIS-Brief (8 items) of ≥21.^13^ This association is independent of sex and seizure frequency and replicates similar findings using the extended BIS (BIS-11).^102^ However, the predictive value of BIS ≥ 21 is poor, with a positive predictive value (PPV) 50% and negative predictive value 69% in the discovery data set.^13^ Higher cut points of BIS increase PPV marginally at the cost of sensitivity. Female sex, social deprivation, a past history of depression, anxiety, personality disorder or recreational drug use (all associated with impulsivity) and status epilepticus have also been suggested as risk factors for psychiatric adverse events to levetiracetam^101,104,105^ and topiramate^106,107^; these factors need prospective validation.

##### Valproate

**Figure 3:**
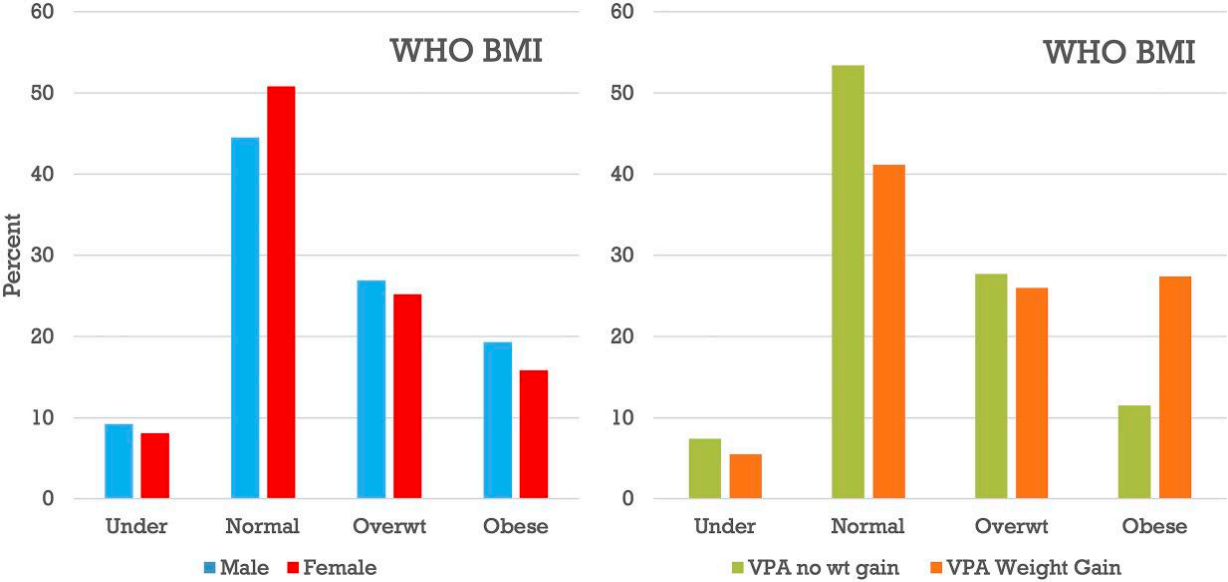
**Association of valproate with obesity in the BIOJUME data set.** WHO categorization of weight in 695 JME patients in the BIOJUME data set by sex (left) and by lifetime exposure to valproate (right). Legend: BMI, body mass index; Overwt, overweight; VPA, valproate; WHO, World Health Organization; wt, weight.

Valproate exposure is associated with obesity in the BIOJUME data set (Fig. 3). We found no relevant univariate or multivariate associations with valproate weight gain in the BIOJUME data set including BIS-Brief score, a measure of trait impulsivity (Table 4). Candidate gene studies suggest that valproate-associated weight gain is associated with (i) variation in satiety and energy homeostasis genes leptin and ankyrin^101^ and (ii) polymorphisms in Cytochrome P450, CYP2C19 and in CD36, PPARγ, GNB3 in Han Chinese.^108-113^ (Fig. 3 and Table 4).

**Table 4:** Multivariate analysis of excessive weight gain in 134 JME patients ever exposed to valproate

Logistic regression of excessive weight gain in valproate exposed JME patients			
Variable	Odds ratio (95% CI)	*Z*	*P*-value
Absence seizure freq	1.45 (0.91–2.32)	1.56	0.118
Myoclonic seizure freq	0.83 (0.52–1.32)	−0.79	0.432
Morning predominant seizures	0.68 (0.28–1.63)	−0.87	0.384
Sex female	1.69 (0.77–3.71)	1.31	0.190
BIS-Brief score ≥21	0.74 (0.34–1.62)	−0.75	0.454
Log_10_ BMI	17.56 (2.96–104.2)	3.15	**0**.**002**

BMI is measured at outcome and therefore not a predictor.

##### Lamotrigine

There is a known HLA predisposition to severe cutaneous reaction to aromatic ASMs (phenytoin, carbamazepine, oxcarbazepine, and lamotrigine),^114^ and these HLA types are more common in certain ancestries such as HLAB*15:02 in Chinese and Southeast Asian; HLAB*24:02 in Han Chinese and possibly other Asian, European and American populations^115,116^; HLAB*31:01 in Japanese; and HLAB*44:03 in Koreans.^114^ Several other candidate loci have been reported in different populations.^117-119^ Independent risk factors such as history of previous cutaneous reactions with another ASM, age < 13 years and polytherapy were identified in retrospective data.^120,121^

## Discussion

We propose a simplified set of mandatory criteria for defining JME that refine and expand those proposed recently by the ILAE Position Paper on definition of IGEs.^5^ However, differently from this position statement, our proposal, besides literature data, is based on previous analysis performed by our group in the large BIOJUME cohort, thus advancing from current definitions resulting from expert consensus to criteria based on ‘ground truth’ clinical data. The criteria that we propose include (i) myoclonic jerks as mandatory seizure type and (ii) generalized EEG abnormalities, in line with the ILAE statement; in addition, we provide as additional criteria that deviate from the ILAE proposal: (iii) absence of a circadian timing for myoclonia not exclusionary for JME; (iv) age of onset ranging from 6 to 40 years consistent with the diagnosis of JME; and (v) intelligence conforming to population distribution. We show the prognostic value, when appropriately analysed, of some variables (such as absence seizures and female sex with regard to ASM resistance), whilst we find that other features (e.g. morning predominance of myoclonia) had no value and may be discarded. When considering prognosis, a simple model is not sufficient to understand the outcome of interest, such as drug response or adverse events. Evidence suggests that, for ASM resistance in JME, female sex is a key stratifying variable overlooked in previous research or unmasked by the avoidance of valproate in females of child-bearing age. A sex-stratified analysis brings into perspective the prognostic roles and therapeutic potential of taking in consideration and making recommendations manipulating stress response, catamenial seizures and photosensitivity in women. Future research should focus on refining, replicating and validating proposed predictive models^122^ using large-scale data sets and meta-analyses and in mapping stratifying factors onto evidence-based interventions and clinical guidelines (Table 5).

**Table 5:** Robust and potential stratifiers in JME

	Robust criteria	Requires validation
Variable features for treatment outcome prediction	Absence seizures Female sex Catamenial seizures Photosensitivity Age of first motor seizure onset <12 years	Stress-triggered seizures in females
Variable features for prediction of selected adverse event to ASM	Lamotrigine rash Ethnicity/HLA status Cross-reaction to ASM Age < 13 years Polytherapy	Levetiracetam psychiatric symptoms Pre-existing psychiatric diagnosis or personality disorder HLA associations Valproate weight gain *LEPR* and *ANK1* polymorphisms *CYP2C19*, *CD36*, *PPAR*γ, and *GNB3*

Existing taxonomy structures do not focus on outcomes; thus, their usefulness for stratified medicine or for clinical trials is limited.^123,124^ When we focus on prognosis, the relevance of definition and classification becomes obvious. In this paper, we propose a set of simplified criteria to define JME aiming to avoid the inductive fallacies characterized by earlier attempts, and our scheme is robust to counterfactual arguments. The debate around the morning occurrence of myoclonic seizures seems moot in light of the finding that lack of circadian pattern probably reflects the co-occurrence of absence seizures and EEG GSWs. An interesting hypothesis that should be tested in patients featuring absence seizures is whether morning predominance could be, at least partially, restored by complete control of this seizure type.

There are enough markers of differential treatment outcome to make a case for a stratified treatment approach in JME. The idea of stratified treatment is novel in epilepsy but commonplace, for example, in cardiology and oncology. In this example, it would entail selecting treatment according to the various prognostic subgroups that each patient falls into guided by a combination of demographic, clinical, neurophysiological, molecular or other biomarker indications. The change from current practice is to consider the management of each prognostic factor separately or in combination as appropriate and according to the relative influence of each factor on the outcome of interest. An analogy might be made with managing cardiovascular disease, simultaneously attending to blood pressure, lipids, exercise and diet. In JME, absence seizures are the strongest stratifying factor with regard to ASM resistance or seizure freedom for both sexes, and this finding has been replicated multiple times.^15^ After absence seizures, it is evident that sex is the major stratifying factor, revealing very strongly elevated odds of ASM resistance conferred by self-report of catamenial and stress-related factors including sleep deprivation, as well as reduced odds of ASM resistance associated with EEG-measured or self-reported photosensitivity, selectively in women. Although menstrual and stress-related triggers have long been recognized,^75,76,125,126^ their influence on *prognosis* in women has not previously been appreciated; this may warrant further research aiming to develop predictive models. Sex-stratified meta-analyses of existing data sets using individual patient data would be helpful to replicate these findings, and prospective studies in inception cohorts are necessary to validate them in real-world practice for JME and other epilepsies. Appropriately validated biomarkers could be incorporated into clinical guidelines and clinical trial designs.

In addition to identifying groups at differential risk of ASM resistance, stratifiers should ideally map onto specific evidence-based interventions. There is an urgent need for clinical trials of novel or existing/adjunctive ASMs stratified on prognostic factors such as the occurrence of absence seizures in JME. Catamenial seizures are also well recognized as a risk factor for ASM resistance; secondary analysis of previous hormonal trials suggests the need for further stratification according to seizure susceptibility at different times during the menstrual cycle.^127^ Although the female-specific prognostic significance of response to stressors including sleep deprivation needs replication, there is a notable lack of good quality evidence for the effectiveness of stress or sleep management strategies in epilepsy.^128-130^ Additionally, whilst lifestyle management improves stress, mental health and sleep, the evidence for sustained effects on primary disease measures is less solid.^131-133^ Pharmacological and non-pharmacological interventions such as cognitive behavioural therapy, mindfulness-based relaxation, yoga, play, biofeedback and exercise should be evaluated according to stress-response profiles.^134^ Connected to lifestyle modification is the seizure refractoriness due to ASM non-compliance (i.e. ‘pseudo-resistance’) that might be linked to the psychological challenges of regulating lifestyle or adhering to prescribed medications or to the impulsivity trait, as recently reported.^135^ This is especially salient in JME where we know that impulsivity is markedly elevated^13,136-138^ and increases general risk for multiple adverse psychosocial consequences related to lifestyle, relationships and behaviour.^139^

In contrast to ASM resistance, there are few validated predictors of ASM adverse events other than known HLA associations with drug-induced immune adverse response.^114^ Behavioural traits and psychiatric history might predict psychiatric adverse events to multiple ASMs^10,15,27,57,86-88^; however, prospective studies are needed to validate these hypotheses. Whilst valproate prescription has rightfully declined in recent years because of teratogenicity, it remains the most effective ASM in JME,^59,98,140^ ensuring its continued role in men. However, one of its main adverse events leading to withdrawal is weight gain, and predicting this risk would be a prioritized patient benefit.

Identifying molecular biomarkers is an alternative approach to prediction than using clinical and demographic variables. Molecular biomarkers are likely to contribute greatly to prognosis in future epilepsy practice. For example, there is evolving evidence that the circulating inflammatory marker HMGB1 may predict seizure severity and drug resistance at epilepsy diagnosis.^141,142^ Similarly, polygenic risk scores will assume an increasing role in enhancing risk prediction models and clinical pathways.^143^ EEG-based prognosis can be limited in resource-poor settings, or when relying on age-related EEG markers (such as GSW before ASM is started, or PPR in adolescence) but the opportunity for that EEG was missed, and the patient is now an adult. The prognostic EEG features reviewed here have current drawbacks in terms of human resources, but these could be solved by AI approaches that learn from high dimensional data sets. A machine learning model predicted drug resistance 2 years before epilepsy patients had failed two ASMs and could predict which patients would fail more than or equal to three ASMs at the time of first ASM prescription.^144^ Whilst we have focused on medical outcomes here, we should not neglect psychosocial outcomes, which typically arise from multiple genetic, early life and stressful life events interacting with adjustment styles and social support.^145^ Detailed lifespan studies or population linkage data sets would be indispensable to generate appropriate psychosocial prognostic models.

## Conclusion

Our paper provides evidence-based diagnostic criteria for JME based on clinical data collected by the BIOJUME Consortium, supplemented by literature review, and it proposes a predictive model of ASM resistance that shows the prognostic value of variables such as absence seizures and female sex in predicting ASM resistance or the relevance of photosensitivity in females in reducing the odds of ASM refractoriness. These findings may assist in clinical practice by helping in the early diagnosis of drug-resistant patients or in the management of their treatment, particularly when drug withdrawal is considered after years of seizure freedom. Our findings can inspire future research either to replicate and validate them, to develop further prediction models in large-scale data sets and to assess whether the stratifying factors that we have outlined can serve to measure intervention-related outcomes and whether they can be meaningfully incorporated in clinical guidelines.

## Supplementary Material

fcad182_Supplementary_Data

## Data Availability

The data supporting the findings and conclusions of this study are available from the corresponding authors upon reasonable request.

## Appendix

BIOJUME Consortium:

Lisa Strug, Naim Panjwani, Fan Lin, Danielle Andrade, Jana Zarubova, Zuzana Šobíšková, Cechovaz, Pracoviste, Michaela Kajsova, Guido Rubboli, Rikke S. Møller, Elena Gardella, Christoph P. Beier, Joanna Gesche, Maria Miranda, Inga Talvik, Pasquale Striano, Alessandro Orsini, Choong Yi Fong, Ching Ching Ng, Kheng Seang Lim, Kaja K. Selmer, Marte Syvertsen, Pronab Bala, Amy Kitching, Kate Irwin, Lorna Walding, Lynsey Adams, Uma Jegathasan, Rachel Swingler, Rachel Wane, Julia Aram, Nikil Sudarsan, Dee Mullan, Rebecca Ramsay, Vivien Richmond, Mark Sargent, Paul Frattaroli, Matthew Taylor, Marie Home, Sal Uka, Susan Kilroy, Tonicha Nortcliffe, Halima Salim, Kelly Holroyd, Khalid Hamandi, Alison McQueen, Dympna Mcaleer, Dina Jayachandran, Dawn Egginton, Bridget MacDonald, Michael Chang, David Deekollu, Alok Gaurav, Caroline Hamilton, Jaya Natarajan Inyan Takon, Janet Cotta, Nick Moran, Jeremy Bland, Rosemary Belderbos, Heather Collier, Joanne Henry, Matthew Milner, Sam White, Michalis Koutroumanidis, William Stern, Mark P. Richardson, Jennifer Quirk, Javier Peña Ceballos, Anastasia, Papathanasiou, Ioannis Stavropoulos, Dora Lozsadi, Andrew Swain, Charlotte Quamina, Jennifer Crooks, Tahir Majeed, Sonia Raj, Shakeelah Patel, Michael Young, Melissa Maguire, Munni Ray, Caroline Peacey, Linetty Makawa, Asyah Chhibda, Eve Sacre, Shanaz Begum, Martin O’Malley, Lap Yeung, Claire Holliday, Louise Woodhead, Karen Rhodes, Rhys Thomas, Shan Ellawela, Joanne Glenton, Verity Calder, John Davis, Paul McAlinden, Sarah Francis, Lisa Robson, Karen Lanyon, Graham Mackay, Elma Stephen, Coleen Thow, Margaret Connon, Martin Kirkpatrick, Susan MacFarlane, Anne Macleod, Debbie Rice, Siva Kumar, Carolyn Campbell, Vicky Collins, William Whitehouse, Christina Giavasi, Boyanka Petrova, Thomas Brown, Catie Picton, Michael O’Donoghue, Charlotte West, Helen Navarra, Seán J. Slaght, Catherine Edwards, Andrew Gribbin, Liz Nelson, Stephen Warriner, Heather Angus-Leppan, Loveth Ehiorobo, Bintou Camara, Tinashe Samakomva, Rajiv Mohanraj, Vicky Parker, Rajesh Pandey, Lisa Charles, Catherine Cotter, Archana Desurkar, Alison Hyde, Rachel Harrison, Markus Reuber, Rosie Clegg, Jo Sidebottom, Mayeth Recto, Patrick Easton, Charlotte Waite, Alice Howell, Jacqueline Smith, Rosie Clegg, Shyam Mariguddi, Zena Haslam, Elizabeth Galizia, Hannah Cock, Mark Mencias, Samantha Truscott, Deirdre Daly, Hilda Mhandu, Nooria Said, Mark Rees, Seo-Kyung Chung, Owen Pickrell, Beata Fonferko-Shadrach, Mark Baker, Amy Whiting, Louise Swain, Kirsty O’Brien, Fraser Scott, Naveed Ghaus, Gail Castle, Jacqui Bartholomew, Ann Needle, Julie Ball, Andrea Clough, Shashikiran Sastry, Charlotte Busby Amit Agrawal, Debbie Dickerson, Almu Duran, Muhammad Khan, Laura Thrasyvoulou, Eve Irvine, Sarah Tittensor, Jacqueline Daglish, Sumant Kumar, Claire Backhouse, Claire Mewies, Julia Aram, Nikil Sudarsan, Dee Mullan, Rebecca Ramsay, Vivien Richmond, Denise Skinner, Mark Sargent, Rahul Bharat, Sarah-Jane Sharman, Arun Saraswatula, Helen Cockerill, David A. Greenberg.

For details of BIOJUME sites, see Supplementary Appendix 1.

## Abbreviations

ASM =: antiseizure medications
BIOJUME =: Biology of Juvenile Myoclonic Epilepsy
BIS =: Barratt Impulsiveness Scale
BMI =: body mass index
CAE =: childhood absence epilepsy
EGTCSA =: epilepsy with generalized tonic–clonic seizures alone
GSW =: generalized spike wave
GTCS =: generalized tonic–clonic seizure
HLA =: human leucocyte antigen
ICD-10 =: International Classification of Diseases 10th Revision
IGE =: idiopathic generalized epilepsy
ILAE =: International League Against Epilepsy
IQ =: intelligence quotient
JME =: juvenile myoclonic epilepsy
NASR =: North American SUDEP Registry
PPR =: photoparoxysmal response
PPV =: positive predictive value
SNOMED-CT =: Systematized Nomenclature of Medicine–Clinical Terms
SUDEP =: sudden unexpected death in epilepsy
TMS-EEG =: transcranial magnetic stimulation–electroencephalography
