# Supplementary material for: Variation in prognosis and treatment outcome in juvenile myoclonic epilepsy: a Biology of Juvenile Myoclonic Epilepsy Consortium proposal for a practical definition and stratified medicine classifications
Source: Brain Commun. 2023 Jun 9;5(3):fcad182. doi: 10.1093/braincomms/fcad182 (PMC10288558; doi:10.1093/braincomms/fcad182)
Supplement: fcad182_Supplementary_Data [file fcad182_supplementary_data.zip › Supplementary Material .docx]

**Supplementary table 1**

| **Diagnosis** | **Mean age of onset, years (range)** | **Initial symptoms** | **Additional symptoms** | **MRI** | **EEG (background)** | **Spikes/**  **polyspikes (freq)** | | **Reference** |
| --- | --- | --- | --- | --- | --- | --- | --- | --- |
| Unverricht-Lundborg | 12.5 (6-17) | Seizures | Invalidating myoclonus/ataxia, no-moderate cognitive impairment | Normal | May be normal, stable | PPR, SW | ^1,2^ | |
| Sialidose 1 | 21 (8-32) | Variable | Gait impairment, ataxia | Normal | Normal | SW | | 3-4 |
| CNL4 (Parry disease) | 30 (25-46) | Seizures | Rapid deterioration and death | Normal | Abnormal | Variable, eg SW/PSW | | ^5,6^ |
| CNL6 (Kufs disease) | 30 (16-62) | Seizures | Dementia, ataxia | Atrophy | Normal to slow/irregular | PPR, SW, multifocal | | ^5,7,8,^ |
| Action myoclonus – renal failure syndrome | 20 (15-25) | Tremor, PML | No cognitive decline, tremor, renal failure | Normal, later atrophy | Progressive slowing | PSW | | ^9,10^ |
| MERRF | 30.1 (0-66) | Ataxia, weakness | Cognitive decline | Normal, later atrophy | Normal to diffuse slowing | SW, PSW, focal spikes | | ^11-13^ |
| Lafora disease | 15 (8-19) | variable | Progressive dementia | normal, later atrophy | Slowing | SW, PSW, focal spikes | | ^14^ |
| Myoclonic astatic epilepsy | 3 (0.7-6) | Several seizure types | Cognitive decline after onset of seizures | Normal | Non-characteristic changes | SW/PSW | | ^15^ |
| Creutzfeld Jakob disease | 60 (vCJD19-39)  (CJD 55-70) | Rapid cognitive decline | Variable | CJD changes | Periodic discharges/ triphasic potentials | no | | ^16^ |
| 15q13.3 microdeletion | 12.3 (7-17) | Absence, GTCS | Moderate cognitive impairment | Normal |  | SW/PSW (2.5-4) | | ^17^ |

Supplementary Table 1. Mimics of Juvenile Myoclonic Epilepsy. SW-spike wave; PSW-polyspike and wave; PPR-photoparoxysmal response.

**Supplementary reference list**

1 - Magaudda A, Ferlazzo E, Nguyen VH, Genton P. Unverricht-Lundborg disease, a condition with self-limited progression: long-term follow-up of 20 patients. Epilepsia. 2006; 47: 860-866. doi: 10.1111/j.1528-1167.2006.00553.x.

2 - Crespel A, Ferlazzo E, Franceschetti S, et al. Unverricht-Lundborg disease. Epileptic Dis. 2016; 18: 28-37. doi: 10.1684/epd.2016.0841.

3 - Canafoglia L, Robbiano A, Pareyson D, et al. Expanding sialidosis spectrum by genome-wide screening: NEU1 mutations in adult-onset myoclonus. Neurology. 2014; 82: 2003-2006. doi: 10.1212/WNL.0000000000000482.

4 - Caciotti A, Melani F, Tonin R, et al. Type I sialidosis, a normosomatic lysosomal disease, in the differential diagnosis of late-onset ataxia and myoclonus: An overview. Molecular genetics and metabolism. 2020; 129: 47-58. doi: 10.1016/j.ymgme.2019.09.005.

5- Nita DA, Mole SE, Minassian BA. Neuronal ceroid lipofuscinoses. Epileptic disorders : international epilepsy journal with videotape. Epileptic Dis 2016; 18(S2): 73-88. doi: 10.1684/epd.2016.0844.

6 - Naseri N, Sharma M, Velinov M. Autosomal dominant neuronal ceroid lipofuscinosis: Clinical features and molecular basis. Clin Genet. 2021; 99: 111-118. doi: 10.1111/cge.13829.

7 - Arsov T, Smith KR, Damiano J, et al. Kufs disease, the major adult form of neuronal ceroid lipofuscinosis, caused by mutations in CLN6. Am J Hum Genet. 2011; 88: 566-573. doi: 10.1016/j.ajhg.2011.04.004.

8 - Berkovic SF, Oliver KL, Canafoglia L, et al. Kufs disease due to mutation of CLN6: clinical, pathological and molecular genetic features. Brain. 2019; 142: 59-69. doi: 10.1093/brain/awy297.

9 - Badhwar A, Berkovic SF, Dowling JP, et al. Action myoclonus-renal failure syndrome: characterization of a unique cerebro-renal disorder. Brain. 2004; 127: 2173-2182. doi: 10.1093/brain/awh263.

10 - Rubboli G, Franceschetti S, Berkovic SF, et al. Clinical and neurophysiologic features of progressive myoclonus epilepsy without renal failure caused by SCARB2 mutations. Epilepsia. 2011; 52: 2356-2363. doi: 10.1111/j.1528-1167.2011.03307.x.

11 - Mancuso M, Orsucci D, Angelini C, et al. Phenotypic heterogeneity of the 8344A>G mtDNA "MERRF" mutation. Neurology. 2013; 80: 2049-2054. doi: 10.1212/WNL.0b013e318294b44c.

12 - Bindu PS, Sonam K, Govindaraj P, et al. Outcome of epilepsy in patients with mitochondrial disorders: Phenotype genotype and magnetic resonance imaging correlations. Clin Neurol Neurosurg . 2018; 164:182-189. doi: 10.1016/j.clineuro.2017.12.010.

13 - Canafoglia L, Franceschetti S, Antozzi C, et al. Epileptic phenotypes associated with mitochondrial disorders. Neurology. 2001; 56: 1340-1346. doi: 10.1212/wnl.56.10.1340.

14 - Turnbull J, Tiberia E, Striano P, et al. Lafora disease. Epileptic Disorders. 2016; 18: 38-62. doi: 10.1684/epd.2016.0842.

15 - Tang S, Pal DK. Dissecting the genetic basis of myoclonic-astatic epilepsy. Epilepsia. 2012; 53: 1303-1313. doi: 10.1111/j.1528-1167.2012.03581.x.

16 - Beier CP, Schulz JB. Prioerkrankungen. In: Brandt C, Diener HC, Gerloff C, editors. Therapie und Verlauf neurologischer Erkrankungen. Stuttgart: Kohlhammer; 2012.

17 - Jähn JA, von Spiczak S, Muhle H, et al. Iterative phenotyping of 15q11.2, 15q13.3 and 16p13.11 microdeletion carriers in pediatric epilepsies. Epilepsy Res. 2014; 108: 109-116. doi: 10.1016/j.eplepsyres.2013.10.001.
