# Supplementary material for: Variation in prognosis and treatment outcome in juvenile myoclonic epilepsy: a Biology of Juvenile Myoclonic Epilepsy Consortium proposal for a practical definition and stratified medicine classifications
Source: Brain Commun. 2023 Jun 9;5(3):fcad182. doi: 10.1093/braincomms/fcad182 (PMC10288558; doi:10.1093/braincomms/fcad182)
Supplement: fcad182_Supplementary_Data [file fcad182_supplementary_data.zip › Supplementary_Appendix_1.docx]

**Supplementary Appendix 1: BIOJUME Consortium**

Sites and site investigators included in the BIOJUME Consortium

| Canada | SickKids Hospital, Toronto | Lisa Strug (PI), Naim Panjwani, Fan Lin |
| --- | --- | --- |
|  | Toronto Western Hospital | Danielle Andrade |
| Czech Republic | Charles University | Jana Zarubova (PI), Zuzana Šobíšková, Cechovaz  Pracoviste, Michaela Kajsova |
| Denmark | Danish Epilepsy Center | Guido Rubboli (PI), Rikke S. Møller, Elena Gardella |
|  | Syddansk Universitet | Christoph P. Beier (PI), Joanna Gesche, Maria  Miranda |
| Estonia | Tallin Children’s Hospital | Inga Talvik (PI) |
| Italy | Commissione Genetica | Pasquale Striano (PI), Alessandro Orsini |
| Malaysia | University of Malaya | Choong Yi Fong (PI), Ching Ching Ng, Kheng  Seang Lim |
| Norway | Vestre Viken Health Trust | Kaja K. Selmer, Marte Syvertsen (Co-PIs) |
| UK | Airedale NHS Foundation Trust | Pronab Bala (PI), Amy Kitching |
|  | Ashford and St. Peter’s  Hospitals NHS Foundation Trust | Kate Irwin (PI), Lorna Walding, Lynsey Adams |
|  | Bradford Teaching Hospitals  NHS Foundation Trust | Uma Jegathasan (PI), Rachel Swingler, Rachel  Wane |
|  | Brighton and Sussex University  Hospitals NHS Trust | Julia Aram (Co-PI), Nikil Sudarsan (Co-PI), Dee  Mullan, Rebecca Ramsay, Vivien Richmond,  Mark Sargent, Paul Frattaroli |
|  | Calderdale and Huddersfield  Foundation Trust | Matthew Taylor (PI), Marie Home, Sal Uka,  Susan Kilroy, Tonicha Nortcliffe, Halima Salim,  Kelly Holroyd |
|  | Cardiff & Vale University Health  Board | Khalid Hamandi (PI), Alison McQueen, Dympna  Mcaleer |
|  | County Durham and Darlington NHS Foundation Trust | Dina Jayachandran (PI), Dawn Egginton |
|  | Croydon Health Services NHS  Trust | Bridget MacDonald (PI), Michael Chang |
|  | Cwm Taf Morgannwg  University Health Board | David Deekollu (Co-PI), Alok Gaurav (Co-PI),  Caroline Hamilton, Jaya Natarajan |
|  | East and North Hertfordshire  NHS Trust | Inyan Takon (PI), Janet Cotta |
|  | East Kent Hospitals University  NHS Foundation Trust | Nick Moran (PI), Jeremy Bland |
|  | East Lancashire Hospitals NHS  Trust | Rosemary Belderbos (PI), Heather Collier,  Joanne Henry, Matthew Milner, Sam White |
|  | Guy's and St Thomas' NHS  Foundation Trust | Michalis Koutroumanidis (PI), William Stern |
|  | King's College Hospital NHS  Foundation Trust | Mark P. Richardson (Co-PI), Jennifer Quirk (Co-  PI), Javier Peña Ceballos, Anastasia  Papathanasiou, Ioannis Stavropoulos |
|  | Kingston Hospital NHS  Foundation Trust | Dora Lozsadi (PI), Andrew Swain, Charlotte  Quamina, Jennifer Crooks |
|  | Lancashire Teaching Hospitals  NHS Foundation Trust | Tahir Majeed (PI), Sonia Raj, Shakeelah Patel,  Michael Young |
|  | Leeds Teaching Hospitals NHS  Trust | Melissa Maguire (Co-PI), Munni Ray (Co-PI),  Caroline Peacey, Linetty Makawa, Asyah  Chhibda, Eve Sacre, Shanaz Begum, Martin O’  Malley |
|  | Manchester University NHS  Foundation Trust | Lap Yeung (Co-PI), Claire Holliday, Louise  Woodhead, Karen Rhodes |
|  | Newcastle upon Tyne Hospitals  NHS Foundation Trust | Rhys Thomas (Co-PI), Shan Ellawela (Co-PI),  Joanne Glenton, Verity Calder, John Davis, Paul  McAlinden, Sarah Francis, Lisa Robson |
|  | NHS Grampian | Karen Lanyon (Co-PI), Graham Mackay (CoPI),  Elma Stephen (Co-PI), Coleen Thow, Margaret  Connon |
|  | NHS Tayside | Martin Kirkpatrick (PI), Susan MacFarlane, Anne  Macleod, Debbie Rice |
|  | North Tees and Hartlepool NHS  Foundation Trust | Siva Kumar (PI), Carolyn Campbell, Vicky Collins |
|  | Nottingham University  Hospitals NHS Trust | William Whitehouse (PI), Christina Giavasi (PI),  Boyanka Petrova, Thomas Brown, Catie Picton,  Michael O'Donoghue, Charlotte West, Helen  Navarra |
|  | Portsmouth Hospitals NHS  Trust | Seán J. Slaght (PI), Catherine Edwards, Andrew  Gribbin, Liz Nelson, Stephen Warriner |
|  | Royal Free London NHS  Foundation Trust | Heather Angus-Leppan (PI), Loveth Ehiorobo,  Bintou Camara, Tinashe Samakomva |
|  | Salford Royal NHS Foundation  Trust | Rajiv Mohanraj (PI), Vicky Parker |
|  | Sandwell & West Birmingham  Hospitals NHS Trust | Rajesh Pandey (PI), Lisa Charles, Catherine Cotter |
|  | Sheffield Children's NHS  Foundation Trust | Archana Desurkar (PI), Alison Hyde, Rachel  Harrison |
|  | Sheffield Teaching Hospitals  NHS Foundation Trust | Markus Reuber (PI), Rosie Clegg, Jo Sidebottom,  Mayeth Recto, Patrick Easton, Charlotte Waite,  Alice Howell, Jacqueline Smith, Rosie Clegg |
|  | Southport and Ormskirk  Hospital NHS Trust | Shyam Mariguddi (PI), Zena Haslam |
|  | St George's University Hospitals  NHS Foundation Trust | Elizabeth Galizia (PI), Hannah Cock, Mark  Mencias, Samantha Truscott, Deirdre Daly,  Hilda Mhandu, Nooria Said |
|  | Swansea University Medical  School and Swansea Bay  University Healthboard | Mark Rees (PI), Seo-Kyung Chung, Owen  Pickrell, Beata Fonferko-Shadrach, Mark Baker |
|  | Taunton & Somerset NHS  Foundation Trust | Amy Whiting (PI), Louise Swain, Kirsty O’Brien |
|  | The Mid Yorkshire Hospitals  NHS Trust | Fraser Scott (Co-PI), Naveed Ghaus (Co-PI), Gail  Castle, Jacqui Bartholomew, Ann Needle, Julie Ball, Andrea Clough |
|  | The Royal Wolverhampton NHS  Trust | Shashikiran Sastry (PI), Charlotte Busby |
|  | The Walton Centre NHS | Amit Agrawal (PI), Debbie Dickerson, Almu  Duran  Foundation Trust |
|  | University Hospitals  Birmingham NHS Foundation  Trust | Muhammad Khan (PI), Laura Thrasyvoulou, Eve  Irvine, Sarah Tittensor, Jacqueline Daglish |
|  | University Hospitals of Derby  and Burton NHS Foundation  Trust | Sumant Kumar (PI), Claire Backhouse, Claire  Mewies |
|  | University Hospitals Sussex  Hospitals NHS Foundation Trust | Julia Aram (Co-PI), Nikil Sudarsan (Co-PI), Dee  Mullan, Rebecca Ramsay, Vivien Richmond,  Denise Skinner, Mark Sargent |
|  | University Hospitals Plymouth  NHS Trust | Rahul Bharat (PI), Sarah-Jane Sharman |
|  | West Suffolk NHS Foundation  Trust | Arun Saraswatula (PI), Helen Cockerill |
| USA | Nationwide Children's Hospital,  Ohio | David A. Greenberg (PI) |
